# Risk factors for morbidity in 1- to 9-day-old dairy calves following caustic paste disbudding

**DOI:** 10.3168/jdsc.2021-0121

**Published:** 2021-09-13

**Authors:** Cassandra N. Reedman, Todd F. Duffield, Trevor J. DeVries, Kerry D. Lissemore, Charlotte B. Winder

**Affiliations:** 1 Department of Population Medicine, University of Guelph, Guelph, ON, N1G 2W1 Canada; 2 Department of Animal Biosciences, University of Guelph, Guelph, ON, N1G 2W1 Canada

## Abstract

Calfhood morbidity is an important issue on commercial dairy operations, with substantial production and welfare impacts. The objective of this observational study was to evaluate potential risk factors for morbidity, including disbudding, disbudding pain control, serum total protein (STP) concentrations, and haptoglobin concentrations in young dairy calves. A total of 140 heifer calves from a commercial dairy farm in southwestern Ontario, Canada, were enrolled at 1 to 9 d of age and followed for 1 wk. Calves were scored at enrollment for several health parameters using a scale from 0 to 3, with 0 representing normal and 3 representing severely abnormal. Calves were only included if they were considered healthy enough to participate using cut points of <3 for rectal temperature and fecal score; ≤2 for nasal discharge, ocular discharge, and cough score; and 1 for ear position, navel score, and joint score. To ensure that only calves that were considered healthy on the baseline day were included, 17 calves were excluded from analysis, resulting in a total of 123 calves. These calves were a part of a disbudding trial, with 100 disbudded using a commercial caustic paste and 23 sham disbudded with a placebo paste. The disbudding paste (commercial or sham) was applied to calves on enrollment day (baseline) with health scoring at baseline and 3 and 7 d later. The following health outcomes were analyzed: diarrhea (a fecal consistency score of ≥2), respiratory disease (a total respiratory score of ≥5), and general sickness [suffering from either diarrhea, respiratory disease, or a navel or joint infection (joint or navel score of ≥2)]. The following variables were assessed for association with the outcomes: STP, inadequate transfer of passive immunity (<5.2 g/dL STP), pain control treatment for disbudding (lidocaine nerve block alone, meloxicam alone, lidocaine nerve block and meloxicam, no pain control, sham), disbudding (disbudded or sham), and haptoglobin concentrations (taken on the baseline day, +3 d and +7 d). We did not detect any relationship between the development of a health outcome of interest (diarrhea, respiratory, or sick) and disbudding or pain control on any of the days following disbudding. However, for every 1 g/dL increase in STP concentration, calves had 0.24 and 0.33 times the odds of suffering from diarrhea or any sickness event, respectively, 3 d after disbudding. At 3 d after disbudding, for every 0.1 mg/mL increase in haptoglobin concentration on that day, calves had 1.34 and 1.20 times the odds of having diarrhea or a general sickness, respectively. These findings suggest that disbudding itself or the pain control method does not appear to influence health after the procedure when healthy calves are disbudded. Calves developing an illness 3 d after disbudding were more likely to have had lower STP concentrations and increased haptoglobin concentrations.

## Highlights

• Disbudding was not detected to be a risk factor for illness.
• Pain control method was not detected to be a risk factor for illness.
• Calves with higher serum total protein values were less likely to get sick 3 d after disbudding.
• Higher haptoglobin values were associated with illness in the week after disbudding.

## Graphical Abstract

**Figure:**
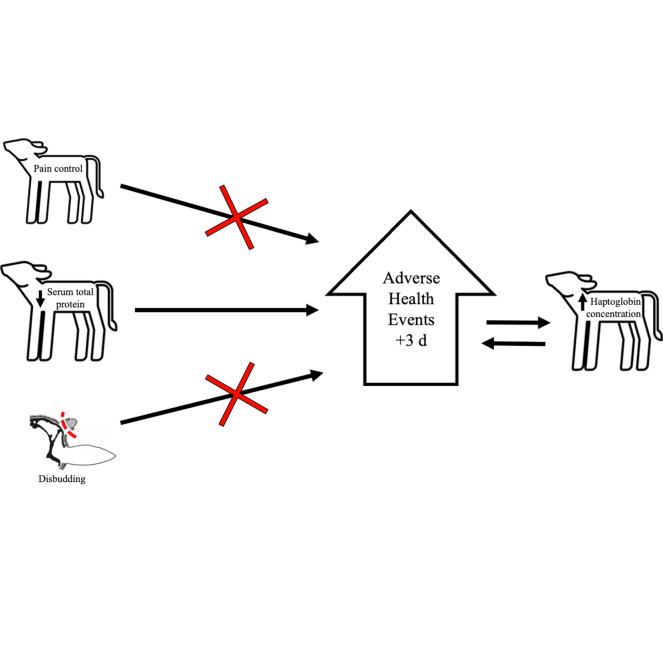
**Summary:** Calfhood morbidity is an important issue in the dairy industry, with substantial production and welfare impacts. The objective of this study was to evaluate potential risk factors for adverse health events in dairy calves following caustic paste disbudding. Our results indicated that calves with higher serum total protein concentrations were less likely to develop an illness in their first week of life compared with calves with lower values, and that increased haptoglobin concentrations were associated with diarrhea and sickness occurrence. Disbudding itself or various pain control treatments were not identified as risk factors for the development of illness.

Calfhood disease has been linked to decreased growth, decreased survival to first calving, and increased age at first calving, and it adversely affects first-lactation milk production (Stanton et al., 2012). Prevalence of calfhood diseases is variable across dairy operations, ranging from 13.7 to 37.2% (NAHMS, 2011; Calderón-Amor and Gallo, 2020). There are many ways to mitigate the risk of adverse health events in calves, including proper colostrum management and reducing environmental stress or stressful events (Beam et al., 2009; Urie et al., 2018; Renaud et al., 2020). Transfer of passive immunity from the dam via colostrum is strongly linked to the subsequent health of the calf (Cuttance et al., 2018; Cortese et al., 2020). Specifically, feeding higher volumes of colostrum is associated with higher serum total protein (**STP**) concentrations and, therefore, reduced odds of inadequate transfer of passive immunity (**ITPI**; Renaud et al., 2020). Lower STP is associated with greater incidence of morbidity and mortality (Urie et al., 2018; Cortese et al., 2020), as well as ITPI associated with increased diarrhea and pneumonia preweaning (Cuttance et al., 2018). Haptoglobin is commonly evaluated as an indicator of inflammation in cattle (Makimura and Suzuki, 1982), which increases following cautery disbudding procedures (Allen et al., 2013) and is associated with increased risk of diarrhea (Chae et al., 2019). Disbudding, particularly without the use of anesthesia or analgesia, is a painful husbandry procedure and has been identified as a risk factor for overall poor calf welfare on dairy operations (Calderón-Amor and Gallo, 2020). As a stressful event for the calf, disbudding can be a risk factor for morbidity in calves when combined with other stressful events (e.g., weaning, nutrition and environmental change), and the reduction of the cumulative effects of such stressors can result in a decrease in morbidity and mortality in calves (Lorenz et al., 2011).

The objective of this study was to evaluate potential risk factors for diarrhea, respiratory disease, or general sickness diagnosed 3 and 7 d following caustic paste disbudding in individually housed calves on a single dairy farm. Associations were tested between health outcomes and disbudding, transfer of passive immunity, serum total protein concentrations, and haptoglobin concentrations (as a measure of inflammation) in young dairy calves for each data collection day. We hypothesized that calves that were disbudded, had ITPI, or had lower STP concentrations would have increased odds of developing diarrhea or pneumonia compared with sham disbudded calves and calves with higher STP concentrations, respectively. We also hypothesized that haptoglobin concentrations would be greater in calves that developed illness following disbudding compared with those that did not.

Data were collected twice weekly between May and August 2018 at a commercial dairy farm in southwestern Ontario, Canada. Individually housed Holstein heifer calves enrolled in this study were part of a randomized controlled trial evaluating effect of pain control strategies on behavioral outcomes (Reedman et al., 2020). Briefly, calves were randomly assigned to 1 of 5 treatment groups consisting of a sham group (sham paste; OmniVet Pharma Inc./Philco Animal Health Inc.), a positive control group (no pain control), a lidocaine cornual nerve block only group (12 mL per calf, lidocaine hydrochloride injection 20 mg/mL, Bimeda-MTC Animal Health Inc.), a meloxicam only group (0.5 mg/kg subcutaneous injection, Metacam 20 mg/mL Solution for Injection, Boehringer Ingelheim), and a group receiving both lidocaine and meloxicam. All calves that were not in the sham control group were disbudded with commercial caustic paste (calcium hydroxide 37.8%, sodium hydroxide 24.9%, Dr Naylor Dehorning Paste, H. W. Naylor Co. Inc.). Sample size (n = 140) justification is described in Reedman et al. (2020).

Data were collected from an individual calf on 3 visits to the farm: baseline (the day the calf was enrolled and disbudded), +3 d, and +7 d. Health was evaluated by the research team by scoring rectal temperature, fecal consistency, cough, navel, ocular discharge, ear position, nasal discharge, and joints. Each parameter was scored on a scale from 0 to 3, with 0 representing normal, 1 representing mildly abnormal, 2 representing moderately abnormal, and 3 representing severely abnormal (McGuirk, 2013; McGuirk and Peek, 2014). Calves were enrolled if they met all of the following (at baseline): <3 for rectal temperature and fecal score; ≤2 for nasal discharge, ocular discharge, and cough score; and 1 for ear position, navel score, and joint score. Calves were then scored on d +3 and +7. On these days, a calf was considered to have respiratory disease when she had a total respiratory score ≥5 [sum of rectal temperature, cough, nasal discharge, and either ocular discharge or ear position (the higher value); McGuirk and Peek, 2014]. Diarrhea was defined as a fecal score of ≥2, and calves were categorized as generally “sick” if they met either the criteria for respiratory disease or diarrhea or had an inflamed navel (navel score of ≥2) or joint (joint score of ≥2). Each health outcome was dichotomized as 1 = unhealthy (respiratory, diarrhea, or sick) and 0 = healthy for each day, and served as the dependent variable in each logistic regression model.

Blood was collected using red top Vacutainer tubes (Becton, Dickinson and Co.) from the jugular vein of each calf on the baseline day when the calves were between 1 and 9 d of age to determine the STP concentration. The STP concentration was used to determine transfer of passive immunity status for each calf, specifically, whether the calf had ITPI (cut-off of 5.2 g/dL; Buczinski et al., 2018). This variable was dichotomized as 1 = ITP (<5.2 g/dL) and 0 = transfer of passive immunity (≥5.2 g/dL). Both ITPI status and STP concentrations served as independent variables in the logistic regression models, and these variables were analyzed separately. Blood samples for haptoglobin were collected on baseline, +3 d, and +7 d. Haptoglobin concentration served as an independent continuous variable in the logistic regression models.

All recorded data were entered into Excel (Microsoft Corp.) and imported into STATA15 (Stata/IC Version 15.1 for Mac, StataCorp). The experimental unit was the calf. Descriptive statistics were reviewed for all variables. Continuous variables (haptoglobin, STP, and age on day of disbudding) were also assessed for normality, linearity, and variation through assessment of outliers and residuals. There was minimal missing data; however, calves with some missing data were still included in the analysis and these missing parameters were reported in the results. Results were considered statistically significant if the *P*-value was <0.05 and the 95% confidence interval did not cross 1 for measures of association (odds ratio).

A mixed effect model was built for each health outcome (diarrhea, respiratory, sickness) with disbudding date included as a random effect. For all dichotomous outcomes (respiratory, diarrhea, and sick) mixed-effect logistic regression models were used with individual models built for both the +3 and +7 d time points. Univariate association of ITPI status, STP, disbudding treatment, and haptoglobin concentrations on the health outcomes were assessed. For +3 d models, baseline haptoglobin concentrations and +3 d haptoglobin concentrations were offered to the model, and for +7 d models, baseline haptoglobin concentrations and +7 d haptoglobin concentrations were offered to the model. Potentially significant variables (*P* < 0.2) from the univariable models and disbudding treatment were kept in the multivariable model for each health outcome at the different time points (pneumonia, diarrhea, and sick) and were further assessed for significance. Calf age on the day of enrollment was offered to each multivariable model and kept if significant (models for diarrhea at +3 d and sickness at +3 d). Model fit was evaluated through the assessment of normality of the best linear unbiased predictors (BLUPs).

Data were also categorized into 2 groups: disbudded calves and sham calves; to investigate the effect of disbudding regardless of the pain control treatment administered. The effect of disbudding on health outcomes was assessed separately from disbudding treatment. Data from the sham calves alone was also investigated to determine whether the pattern of increased sickness across the days (baseline, +3 d, and +7 d) was correlated with disbudding or age.

In total, data were collected on 140 heifer calves; however, 17 calves were excluded from this analysis because 15 had fecal scores of 2 on the baseline day and 2 had a navel score of 2 on the baseline day and, therefore, for the purposes of this analysis, were considered sick on this day. This resulted in a total of 123 calves included in this analysis. On the day of enrollment, 100 calves were disbudded (23 in the no pain control group, 26 receiving meloxicam alone, 24 receiving lidocaine alone, and 27 receiving both lidocaine and meloxicam) and 23 were sham disbudded. All samples and measurements that were collected were used for analysis. Table 1 includes information on the number of calves with missed observations on each day. Calf age ranged from 1 to 9 d on the day of disbudding, with a mean age of 3.7 ± 0.09 d. The percent of calves suffering from the 3 illnesses of interest (respiratory, diarrhea, and sick) on each day of data collection is given in Table 1.

**Table 1:** Raw data of calves suffering from illnesses of interest

Health outcome	Time period	Calves suffering from an illness (n = 123) [% (no./no.)]
Fecal illness/diarrhea^1^	Baseline	0 (0/123)
	+3 d	28.9 (35/121)^2^
	+7 d	53.7 (66/123)
Respiratory illness^3^	Baseline	0 (0/123)
	+3 d	6.5 (8/123)
	+7 d	0.8 (1/123)
Sick^4^	Baseline	0 (0/123)
	+3 d	34.1 (42/123)
	+7 d	53.7 (66/123)

1 Calves with a fecal consistency score of 2 or 3 on a scale from 0 to 3.

2 Data were not collected on all 123 calves for these outcomes and therefore were not available for analysis.

3 Calves with a respiratory score ≥5 [calculated by summing rectal temperature, cough, nasal discharge, and either ocular discharge or ear position (the higher value) scores, which were all scored on a scale from 0 to 3].

4 Calves suffering from diarrhea, a respiratory illness, or a navel or joint infection (score of 2 or 3 on a scale from 0 to 3).

The percentage of calves either suffering from diarrhea or that were sick increased over the week they were followed (Table 1). The final multivariable model outputs for calves suffering from diarrhea at +3 and +7 d and calves suffering from any sickness at +3 d are described in Table 2, Table 3, respectively. The model evaluating sickness at +7 d was identical to that evaluating diarrhea at this time because every sick calf on this day was sick due to diarrhea; therefore, this model was not included. None of the predictor variables of interest (ITPI, STP, day, haptoglobin, disbudding treatment, disbudding, age) were statistically significant in any of the univariable or multivariable models constructed for respiratory illness on +3 or +7 d after disbudding, most likely due to the very small number of respiratory illness cases observed in this study.

**Table 2:** Outputs from both final multivariable mixed-effect logistic regression models of calves suffering from diarrhea (one model on +3 d after disbudding and one model on +7 d after disbudding) with a random effect for date of disbudding

Item	Odds ratio	95% CI	*P*-value
+3 d relative to disbudding			
Baseline haptoglobin (mg/mL)	1.17	0.88–1.55	0.27
+3 d haptoglobin (mg/mL)	1.34	1.09–1.64	0.006
STP^1^ (g/dL)	0.24	0.07–0.82	0.023
Age on day of disbudding (d)	1.88	1.25–2.82	0.002
Sham^2^	7.23	0.92–56.9	0.06
No pain control^2^	1.56	0.19–12.8	0.68
Lidocaine^3^	3.93	0.59–25.9	0.16
Meloxicam^4^	1.73	0.25–11.9	0.58
Lidocaine and meloxicam^5^	Referent	—	—
+7 d relative to disbudding			
Baseline haptoglobin (mg/mL)	0.91	0.75–1.09	0.31
+7 d haptoglobin (mg/mL)	1.41	0.97–2.03	0.068
STP (g/dL)	0.61	0.32–1.16	0.13
Sham^2^	0.56	0.17–1.87	0.34
No pain control^2^	0.87	0.25–2.96	0.82
Lidocaine^3^	0.63	0.19–2.09	0.45
Meloxicam^4^	0.45	0.14–1.43	0.18
Lidocaine and meloxicam^5^	Referent	—	—

1 STP = serum total protein.

2 n = 23 calves.

3 n = 24 calves.

4 n = 26 calves.

5 n = 27 calves.

**Table 3:** Output from final multivariable mixed-effect logistic regression models of calves suffering from any sickness +3 d after disbudding (diarrhea, respiratory illness, a navel or joint infection) with a random effect for date of disbudding

Item	Odds ratio	95% CI	*P*-value
Baseline haptoglobin (mg/mL)	1.16	0.92–1.47	0.20
+3 d haptoglobin (mg/mL)	1.20	1.03–1.40	0.023
STP^1^ (g/dL)	0.33	0.13–0.83	0.019
Age on day of disbudding (d)	1.39	1.04–1.85	0.024
Sham^2^	3.72	0.78–17.8	0.099
No pain control^2^	1.45	0.28–7.49	0.65
Lidocaine^3^	3.04	0.69–13.4	0.14
Meloxicam^4^	1.95	0.44–8.67	0.38
Lidocaine and meloxicam^5^	Referent	—	—

1 STP = serum total protein.

2 n = 23 calves.

3 n = 24 calves.

4 n = 26 calves.

5 n = 27 calves.

Both STP and ITPI were evaluated separately in univariable and multivariable models of the health outcomes of interest due to correlation issues. Values of STP ranged from 4.3 to 7.9 g/dL with a mean (±SD) of 5.81 ± 0.66 g/dL. Because of the small number of calves suffering from ITPI (19/123), STP was chosen for inclusion in the final models for diarrhea and sickness (Table 2, Table 3, respectively). For every 1 g/dL increase in STP concentrations, calves had 0.24 and 0.33 times the odds of suffering from diarrhea or sickness, respectively, +3 d after disbudding (Table 2, Table 3, respectively). Haptoglobin concentrations on the corresponding day were significant in the final models for diarrhea and sickness on +3 d, but not shown to be significant in any of the models assessing respiratory illness. Baseline haptoglobin concentrations were kept in every model to control for any differences between calves (Table 2, Table 3). Haptoglobin concentrations ranged from 0.06 to 1.68 mg/mL, with a mean (±SD) value of 0.224 ± 0.25 mg/mL across all days (baseline: 0.09–1.45 mg/mL, mean = 0.203 ± 0.21 mg/mL; +3 d: 0.06–1.68 mg/mL, mean = 0.293 ± 0.33 mg/mL; +7 d: 0.07–1.5 mg/mL, mean = 0.190 ± 0.18 mg/mL). For every 0.1 mg/mL increase in haptoglobin concentrations +3 d after disbudding, calves had 1.34 and 1.20 times the odds of suffering from diarrhea or general sickness on the corresponding day, respectively (Table 2, Table 3).

In this study, we investigated the relationships between disbudding, ITPI, STP, haptoglobin concentrations, and health outcomes of young dairy heifer calves. Similar to past research, our results demonstrated that calves are more likely to get sick, particularly with diarrhea, as they grow older (Rocha et al., 2016; Urie et al., 2018). Similar to past work, we also noted that calves with lower STP concentrations were more likely to develop an illness in their first week of life than those with higher values (Urie et al., 2018; Cortese et al., 2020). We also detected that 3 d after disbudding (when calves were between 4 and 12 d old) there was a positive association between haptoglobin concentrations and the occurrence of diarrhea or sickness in calves on the corresponding day. However, because of study limitations that resulted in not sampling for haptoglobin every day, there was an issue of temporality, and we cannot comment on whether concentrations began to increase before the development of an illness or vice versa. It is important to note another limitation to this study; this sample size was calculated based on a randomized controlled trial and, therefore, this study was likely not adequately powered to detect an association between health and disbudding.

Although both diarrhea and respiratory illness are common diseases of preweaning calves, some researchers have reported that digestive diseases occur more frequently than respiratory diseases (NAHMS, 2011; Urie et al., 2018). We observed this as well; very few calves developed respiratory disease over the course of the week that they were followed, whereas more than half of the calves developed diarrhea by 7 d after disbudding. The calves enrolled in the trial were all considered healthy before enrollment. Calves have been reported to have higher incidences of morbidity when they are experiencing stress from environmental factors (Urie et al., 2018; Calderón-Amor and Gallo, 2020) as well as accumulating multiple stressful events such as weaning and painful procedures (Lorenz et al., 2011).

Disbudding is considered a stressful and painful procedure for a calf (Stock et al., 2013). Few studies have examined the effect of disbudding, or pain control during disbudding, on the subsequent health of a calf. In an observational study performed in Chile, no association was detected between pain control for disbudding and adverse health events in calves (Calderón-Amor and Gallo, 2020). However, few farms (2/29) in that work used any form of pain control for disbudding. Although more calves in our study developed an illness as more time passed after disbudding, the pain control treatment (of the 5 previously mentioned) that the calves were assigned to did not have a detectable effect on risk of development of an illness, which could be due to a lack of power to detect this. In addition, disbudding itself did not have a detectable effect on these health outcomes (potentially also due to a lack of power). When comparing the sham disbudded calves to the disbudded calves, we observed the same pattern of increased adverse health events over time, indicating that the development of our health outcomes was likely related to the age of the calf (the older they get the more likely they are to develop an illness) rather than to the disbudding event. Similar to the results reported by Lorenz et al. (2011), the stress of disbudding alone may not be a sufficient cause for morbidity in calves; however, when several stressful events occur around the same time, the cumulative effects can lead to an increased risk of morbidity. Further research in this area (including evaluating farm-level factors) could help to determine whether the stress associated with the disbudding procedure affects the health of the calf.

Haptoglobin is an acute phase protein often used as an indicator of inflammation in cattle (Makimura and Suzuki, 1982), and it has been reported to increase from basal values around 12 h up to 3 d after cautery disbudding in calves (Allen et al., 2013). Similar to research examining diarrhea in weaned calves (Chae et al., 2019), we showed that haptoglobin concentrations had a positive relationship with adverse health outcomes (diarrhea and sickness) in this study; however, we only detected this 3 d after disbudding, with no association detected 7 d after. In contrast, Rocha et al. (2016) did not detect differences in haptoglobin concentrations in calves with diarrhea versus healthy calves. Although our study was not powered to detect a difference in haptoglobin concentrations between healthy and unhealthy calves, we had a larger sample size than Rocha et al. (2016), which may explain this discrepancy. However, the current study's lack of power could also explain why we were unable to detect an association between haptoglobin concentrations and illness 7 d after disbudding.

Concentrations of STP can be used to determine whether a calf has received adequate transfer of passive immunity; however, this metric has also been identified to have an association with morbidity in calves, with lower STP concentrations being associated with increased morbidity (Cuttance et al., 2018; Urie et al., 2018; Cortese et al., 2020). We detected the same relationship between STP concentrations and morbidity in calves; however, we only detected this association 3 d after disbudding or baseline when calves were between 4 and 12 d old. At this time (+3 d), we also determined that age on baseline day was associated with increased morbidity, with older calves having increased odds of suffering from diarrhea or sickness on this day. This result was most likely due to the general association between age and illness—as calves grow older, they are more likely to develop an illness (Rocha et al., 2016; Urie et al., 2018); therefore, the older calves on this day would be more likely to be sick or have developed an illness. Our lack of significant results 7 d after disbudding could be due to a lack of power in this study to detect these differences. Although past researchers have reported the binary metric of ITPI to be useful as a farm-level indicator of colostrum management (Beam et al., 2009; Windeyer et al., 2014; Renaud et al., 2020), very few calves enrolled in this study were suffering from ITPI and, therefore, the study was underpowered to properly evaluate this predictor.

Overall, we detected no effect of either caustic paste disbudding in 1- to 9-d-old dairy calves or pain control protocol on the health of the calf in the week following the procedure when healthy calves were disbudded. However, we did detect a relationship between increased haptoglobin concentrations and increased risk of diarrhea and sickness in calves, which further supports the association between STP concentration and the subsequent health of the animal in the first week of life.
