# Erratum to “Risk factors for morbidity in 1- to 9-day-old dairy calves following caustic paste disbudding” (JDS Commun. 2:376–380)

**DOI:** 10.3168/jdsc.2022-3-2-167

**Published:** 2022-03-16

**Authors:** Cassandra N. Reedman, Todd F. Duffield, Trevor J. DeVries, Kerry D. Lissemore, Charlotte B. Winder

The animal ethics statement was missing from this paper. The following statement should be included: “Use of animals and all methods for this study were approved by the University of Guelph Animal Care Committee (AUP#4001) in compliance with animal use guidelines of the *Canadian Council on Animal Care* (1993).”
